# ADHD and Decision Paralysis: Overwhelm in a World of Choices

**DOI:** 10.1192/j.eurpsy.2025.406

**Published:** 2025-08-26

**Authors:** B. A. Oroian, P. Nechita, A. Szalontay

**Affiliations:** 1 “Grigore T. Popa” University of Medicine and Pharmacy Iasi; 2 “Socola” Institute of Psychiatry Iasi, Iasi, Romania

## Abstract

**Introduction:**

Decision paralysis, defined as the inability to make decisions due to overwhelming options or uncertainty, is an often overlooked symptom of Attention-Deficit/Hyperactivity Disorder (ADHD). Individuals with ADHD frequently struggle with executive dysfunction, making it difficult to prioritize tasks, evaluate choices, and make timely decisions. Despite its significance, decision paralysis remains under-researched, particularly in terms of its impact on daily functioning and quality of life.

**Objectives:**

This study aims to assess the prevalence and severity of decision paralysis in adults with ADHD, explore its relationship with executive dysfunction, and analyze its impact on various life outcomes such as career performance, interpersonal relationships, and overall well-being.

**Methods:**

A total of 50 adults diagnosed with ADHD participated in this study. Self-report measures, including the Decision-Making Competence (DMC) scale and the ADHD Executive Dysfunction Questionnaire (AEDQ), were administered to assess participants’ decision-making difficulties, indecision, and executive functioning. Additional data were collected on life satisfaction, perceived stress levels, and the degree of daily functional impairment due to decision paralysis.

**Results:**

The results indicate that 82% of participants reported frequent difficulties with decision-making, with 68% indicating that decision paralysis significantly affected their work performance. Decision paralysis was strongly correlated with executive dysfunction scores and was a significant predictor of reduced life satisfaction and increased perceived stress. Additionally, 74% of participants reported that indecision contributed to delays or avoidance in making important life choices, such as career changes or financial decisions, leading to long-term dissatisfaction. Notably, 58% of participants experienced decision paralysis at least once a week, with 35% reporting daily occurrences. Furthermore, 61% of participants indicated that decision paralysis led to missed opportunities in both personal and professional contexts, contributing to feelings of regret and frustration.

**Conclusions:**

This study highlights the widespread impact of decision paralysis in adults with ADHD, significantly affecting both personal and professional domains. The strong correlation between decision paralysis and executive dysfunction suggests that addressing this symptom could be critical in improving quality of life for individuals with ADHD. Further research is needed to explore the development of specific interventions targeting decision paralysis.

**Disclosure of Interest:**

None Declared

